# Major Effect of Hydrogen Peroxide on Bacterioplankton Metabolism in the Northeast Atlantic

**DOI:** 10.1371/journal.pone.0061051

**Published:** 2013-04-12

**Authors:** Federico Baltar, Thomas Reinthaler, Gerhard J. Herndl, Jarone Pinhassi

**Affiliations:** 1 Centre for Ecology and Evolution in Microbial Model Systems (EEMiS), Linnaeus University, Kalmar, Sweden; 2 Department of Marine Biology, University of Vienna, Vienna, Austria; 3 Department of Biological Oceanography, Royal Netherlands Institute for Sea Research (NIOZ), Den Burg, The Netherlands; Universidade Federal do Rio de Janeiro, Brazil

## Abstract

Reactive oxygen species such as hydrogen peroxide have the potential to alter metabolic rates of marine prokaryotes, ultimately impacting the cycling and bioavailability of nutrients and carbon. We studied the influence of H_2_O_2_ on prokaryotic heterotrophic production (PHP) and extracellular enzymatic activities (i.e., β-glucosidase [BGase], leucine aminopeptidase [LAPase] and alkaline phosphatase [APase]) in the subtropical Atlantic. With increasing concentrations of H_2_O_2_ in the range of 100–1000 nM, LAPase, APase and BGase were reduced by up to 11, 23 and 62%, respectively, in the different water layers. Incubation experiments with subsurface waters revealed a strong inhibition of all measured enzymatic activities upon H_2_O_2_ amendments in the range of 10–500 nM after 24 h. H_2_O_2_ additions also reduced prokaryotic heterotrophic production by 36–100% compared to the rapid increases in production rates occurring in the unamended controls. Our results indicate that oxidative stress caused by H_2_O_2_ affects prokaryotic growth and hydrolysis of specific components of the organic matter pool. Thus, we suggest that oxidative stress may have important consequences on marine carbon and energy fluxes.

## Introduction

Reactive oxygen species such as hydrogen peroxide (H_2_O_2_) are known to commonly cause oxidative stress in marine organisms (see [1] for review), and therefore, might have the potential to alter metabolic rates of marine prokaryotes, ultimately impacting the cycling and bioavailability of nutrients and organic carbon. H_2_O_2_ commonly occurs in marine and freshwater environments where it can be found in concentrations as high as 10 µM [2], although in open oceans it usually varies between <10–440 nM [3], with concentrations up to 1450 nM in Antarctic waters during autumn and winter periods [4]. Oceanic H_2_O_2_ concentrations generally decrease with depth; with concentrations up to 15 nM and 6 nM at 100 m depth and in the bathypelagic zone, respectively [5]. In the subtropical North Atlantic (our study region), H_2_O_2_ concentrations show a surface maximum of 75–220 nM, followed by a sharp decline at the shallow thermocline of ∼40 m depth [6]. In deeper waters in this region H_2_O_2_ concentrations are relatively stable, varying between 5–10 nM [6] which is in agreement with concentrations of up to 6 nM found at 150–5500 m in the subtropical North Pacific [5].

H_2_O_2_ formation is traditionally attributed to photo-oxidation of chromophoric dissolved organic matter by molecular oxygen [7], [8]. However, there is growing evidence that biological production could also be an important source of H_2_O_2_. Dark production of H_2_O_2_ has been found in unfiltered water of the coastal Mediterranean, the Red Sea and the Baltic Sea [9] and in surface waters of the Sargasso Sea [10]. Moreover, overnight production of H_2_O_2_ has been detected during in situ studies in the Atlantic [11], [12] and the Pacific [13]. Yuan and Shiller [13] suggested that biological H_2_O_2_ production might control the in situ H_2_O_2_ concentrations measured in the Northwest Pacific. The detection of H_2_O_2_ concentrations up to 6 nM at 5000 m depth was interpreted as an indication of its biological origin at depth since the short half-life of H_2_O_2_ makes it highly unlikely that photochemically produced H_2_O_2_ reaches this depth [5].

Recently, in situ H_2_O_2_ concentrations were found to be close to a steady state between dark production and decay in samples from depths of ≥10 m, suggesting that the H_2_O_2_ at those depths could be maintained primarily by particle-associated dark production and not by abiotic photochemical processes [14]. These authors suggested that extracellular H_2_O_2_ could be a common byproduct of biological processes (as is intracellular H_2_O_2_), or a product of a specific biochemical process, indicating that H_2_O_2_ production rates might vary with environmental factors and species composition. Interestingly, it was recently hypothesized that abiotic processes such as photoxidation are able to produce peroxides (i.e., hydroperoxides associated with organic matter) that might inhibit bacterial activity by singlet oxygen transfer from phytodetritus to particle-attached bacteria [15]. The oxidative stress provoked by such particle-associated production of reactive oxygen species could be particularly relevant in microhabitats and in the deep ocean due to the preferential particle-related life strategy of dark ocean prokaryotes [16], [17], [18], [19].

H_2_O_2_ can readily diffuse across cytoplasmic membranes, influencing biological processes and damaging cellular constituents [20], [21], [22]. The damage includes formation of hydroxyl radicals inside the cell that might inhibit collagen gelation, modify amino acid residues of proteins and react with cell components, thereby producing organic peroxides which can attack DNA [23], [24], [25], [26]. Lipid peroxides are formed when hydroxyl radicals interact with lipids, affecting the functioning of cell membranes, membrane-bound enzymes and other macromolecules [27]. Superoxide radicals and peroxides may also bind to DNA and alter or break the double helix structure [28].

Concentrations of ∼100 nM H_2_O_2_ influenced bacterioplankton by strongly reducing their abundance in microcosm experiments with water from the Gulf of Mexico [29], and causing oxidative stress in bacteria in Mediterranean waters [30]. In lake waters, the same H_2_O_2_ concentration (100 nM H_2_O_2_) caused a 40% reduction of the prokaryotic heterotrophic production in Lac Cromwell [22], and H_2_O_2_ concentrations of 2000–3000 nM effectively inhibited prokaryotic production in Lake Fiolen [31]. Moreover, H_2_O_2_ is an effective chemical algicide limiting cyanobacterial and green algal growth [32], [33]. If H_2_O_2_ influences bacterioplankton, then H_2_O_2_ could indirectly affect the physico-chemical characteristics of the environment (e.g., inorganic and organic nutrient quality and quantity, CO_2_ fixation and respiration) concomitantly affecting microbial community composition.

Despite the ubiquitous presence of reactive oxygen species such as H_2_O_2_ in the ocean and the crucial role played by marine prokaryotes in the marine biogeochemical cycles, the effect of the oxidative stress caused by reactive oxygen species on the metabolic rates of marine bacterioplankton and its ecological implications remain basically unknown. Here we studied the potential influence of H_2_O_2_ on prokaryotic heterotrophic production and extracellular enzymatic activities (β-glucosidase, leucine aminopeptidase and alkaline phosphatase) in the water column of the subtropical Atlantic. Our results suggest that H_2_O_2_ can have substantial effects on prokaryotic metabolism from the epipelagic to bathypelagic waters, which in turn might have profound implications on marine carbon cycling.

## Methods

### Ethics Statement

No specific permits were required for the described field studies. Sampling locations are not privately-owned or protected and sampling did not involve endangered or protected species.

### Study Site and Experimental Setup

**Figure 1:**
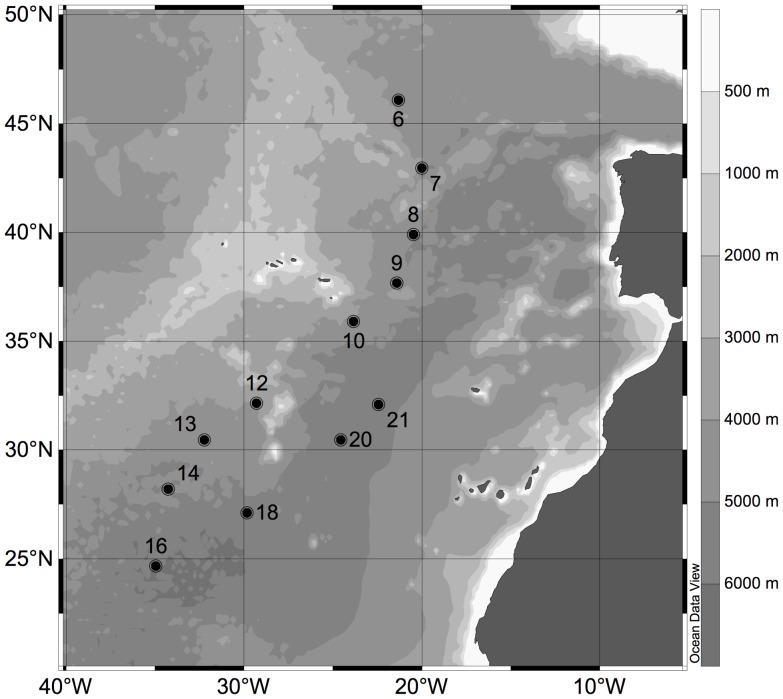
Locations where hydrogen peroxide enrichment experiments were carried out during the MEDEA cruise in October-November 2011.

Two different sets of experiments were conducted with water collected in the subtropical northeast Atlantic Ocean during the MEDEA-I cruise with the RV *Pelagia* in October-November 2011 (Fig. 1). Seawater was collected from the bathypelagic in the core of the North Atlantic Deep Water (NADW, 2700–2800 m depth), the mesopelagic in the oxygen minimum layer (OML, 500–990 m depth) and the epipelagic at the base of the euphotic layer (100 m depth) using 25-L Niskin bottles mounted on a CTD (conductivity-temperature-depth) rosette sampler (Table S1).

In the first set of experiments, 100, 500 and 1000 nM of H_2_O_2_ was added to seawater in duplicate 50 ml sterile conical tubes (Greiner Bio One). Blanks with no H_2_O_2_ addition were simultaneously done. On these samples, triplicate measurements of extracellular enzymatic activity (EEA) were performed immediately after H_2_O_2_ addition as explained below. These experiments were conducted at 9 stations (Stn. 6–16; see Fig. 1). The second set of experiments was carried out at 3 consecutive stations (Stn. 18–21; see Fig. 1) by adding different concentrations of H_2_O_2_ (50, 250, 500 nM at Stn. 18, and 10, 50, 100 nM at Stn. 20–21) to duplicate 1-L bottles containing water from the OML and 100 m depth. Amended samples were incubated together with duplicate unamended controls, in the dark at in situ temperature for 24 h. In this second set of experiments, EEAs were measured in triplicates at the beginning of the experiment (0 h) and after 24 h. Additionally, prokaryotic heterotrophic production (PHP) was measured at the start of the experiment, after 6 h and 24 h. The concentration of H_2_O_2_ stock solutions was determined daily prior to adding H_2_O_2_ to the samples using a spectrophotometer and the molar absorptivity of 38.1±1.4 M^−1^ cm^−1^ at 240 nm [34]. Measurements of in situ H_2_O_2_ concentrations were attempted but failed due to an error in the preparation of the working solutions that were brought to the cruise.

### Measurements of Prokaryotic Extracellular Enzymatic Activity (EEA)

The hydrolysis of the fluorogenic substrate analogs 4-methylcoumarinyl-7-amide (MCA)-L-leucine-7-amido-4-methylcoumarin, 4-methylumbelliferyl (MUF)-phosphate and MUF-β-D-glucoside was measured to estimate the potential hydrolytic activity of leucine aminopeptidase (LAPase), alkaline phosphatase (APase) and β-glucosidase (BGase), respectively [35]. All chemicals were obtained from Sigma. The procedure was followed as described previously [19], [36]. Briefly, EEA was determined after substrate addition and incubation using a spectrofluorometer (Fluorolog-3) with a microwell plate reader (MicroMax 384, Horiba) at an excitation and emission wavelength of 365 and 445 nm, respectively. Samples (300 µl) were incubated in the dark at in situ temperature for 3–24 h. The linearity of the increase in fluorescence over time was checked on sets of samples incubated for 24 to 48 h, resulting in the same hydrolytic rates h^–1^. Subsamples without substrate additions served as blanks to determine the background fluorescence of the samples. Previous experiments showed insignificant abiotic hydrolysis of the substrates [37], [38], [39]. The fluorescence obtained at the beginning and the end of the incubation was corrected for the corresponding blank. This increase in fluorescence over time was transformed into hydrolysis rates using standard curves established with different concentrations of the fluorochromes MUF and MCA added to 0.2 µm filtered sample water. A final substrate concentration of 10 µmol l^–1^ for BGase, 100 µmol l^–1^ for APase and 500 µmol l^–1^ for LAPase was used. These concentrations have been previously determined as saturating substrate concentrations [36], i.e., resulting in maximum hydrolysis rates. Consequently, the EEAs given throughout the paper represent potential hydrolysis rates. The substrates used in this study were previously shown to be unaffected by H_2_O_2_ [40] thus, excluding the possibility of abiotic artifacts due to the added H_2_O_2_.

### Prokaryotic Heterotrophic Production (PHP)

Bulk PHP was measured following the centrifugation method [41]. Triplicate 1 ml live-samples and TCA-killed blanks (5% final concentration) with 10 nM [*^3^*H]-leucine (final concentration, specific activity 140 Ci mmol^−1^; Perkin Elmer) were incubated in temperature-controlled incubators in the dark at *in situ* temperature for 3–24 h. Incubation times were consistent for EEA and PHP incubations, and similar to those used in previous studies in the same region [18], [19], [36]. Incubations were terminated by adding TCA to a final concentration of 5% and live-samples and blanks were centrifuged at 12,000 *g* for 10 min. After aspirating the water, 1 ml of 5% TCA was added followed by a second round of centrifugation. Subsequently, the water was aspirated again, the vials dried and scintillation cocktail (1 ml Canberra-Packard Ultima-Gold) was added. After 18 h, the samples and blanks were counted in a liquid scintillation counter (Tri-Carb 3100TR, Perkin Elmer). The mean disintegrations per minute (DPM) of the TCA-fixed blanks were subtracted from the mean DPM of the respective samples, and the resulting DPM converted into leucine incorporation rates.

## Results

### Effect of H_2_O_2_ Concentration on EEA in the Northeast Atlantic Water Column

In the first set of H_2_O_2_ enrichment experiments (i.e., 100, 500 and 1000 nM), LAPase exhibited the highest rates among the EEA measured followed by APase and BGase (Wilcoxon rank sum test, p<0.0001) (Table 1 and Table S2). Of all the extracellular enzymes tested, LAPase decreased most pronouncedly with depth from the 100 m horizon to the NADW (ca. >4 fold) while APase decreased only by about 50% from 100 m depth to the NADW (Table 1). In contrast to LAPase and APase, BGase remained stable with depth. Throughout the study area, a negative effect of H_2_O_2_ on all the measured EEAs was observed in the epi-, meso- and bathypelagic waters; BGase was more inhibited by H_2_O_2_ than APase and LAPase (Wilcoxon rank sum test, p = 0.0002) (Table 1). On average, H_2_O_2_ reduced BGase rates to 40.3 to 61.9% at all depths, depending on the H_2_O_2_ concentration added. The strongest BGase reduction was found in the 500 nM H_2_O_2_ treatment at all depths. The lowest concentration of H_2_O_2_ used in this first set of experiments (i.e., 100 nM) reduced BGase rates between 20.7–50.5% (Table 1). In contrast, APase and LAPase were much less affected by H_2_O_2_. The strongest APase reduction occurred at 100 m depth, where the addition of 500 and 1000 nM H_2_O_2_ inhibited APase by 19.7 and 23%, respectively (Table 1). In deeper waters, APase was reduced by less than 12.7%. On average, LAPase was never inhibited more than 11% in all depth layers (Table 1).

**Table 1:** Average (±SD) response in the microbial extracellular enzymatic activities (EEAs), to different H_2_O_2_ concentrations in the experiments done with epi- meso- and bathypelagic waters at Stn. 6–16 (n = 9).

Zone	Depth (m)	[H_2_O_2_]	LAPase (nM h^−1^)	APase (nM h^−1^)	BGase (nM h^−1^)	Inh LAPase (%)	Inh APase (%)	Inh BGase (%)
100 m	100	0	7.03 (2.8)	0.76 (0.4)	0.10 (0.06)			
		100	6.65 (2.6)	0.73 (0.3)	0.05 (0.03)	5.4	4.3	50.5
		500	6.50 (2.5)	0.61 (0.2)	0.04 (0.05)	7.5	19.7	61.9
		1000	6.40 (2.4)	0.59 (0.2)	0.05 (0.05)	8.8	23.0	50.7
OML	619–990	0	2.61 (1.2)	0.53 (0.1)	0.10 (0.04)			
		100	2.50 (1.2)	0.50 (0.1)	0.08 (0.03)	4.3	5.7	20.7
		500	2.41 (1.1)	0.50 (0.1)	0.06 (0.03)	7.5	5.4	41.4
		1000	2.39 (1.0)	0.50 (0.1)	0.07 (0.04)	8.3	5.9	29.0
NADW	2700–2800	0	1.66 (1.5)	0.43 (0.1)	0.10 (0.07)			
		100	1.57 (1.4)	0.38 (0.1)	0.06 (0.06)	5.4	12.7	40.3
		500	1.48 (1.3)	0.38 (0.1)	0.05 (0.03)	11.0	11.5	54.0
		1000	1.52 (1.4)	0.39 (0.1)	0.06 (0.03)	8.3	9.8	40.6

Also shown is the corresponding percentage of EEA inhibited (Inh) compared to the control without amendment of H_2_O_2_. LAPase: leucine aminopeptidase, APase: alkaline phosphatase, BGase: β-glucosidase. OML: oxygen minimum layer, NADW: North Atlantic Deep Water.

### Temporal Variation of the H_2_O_2_ Impact on PHP and EEA

**Figure 2:**
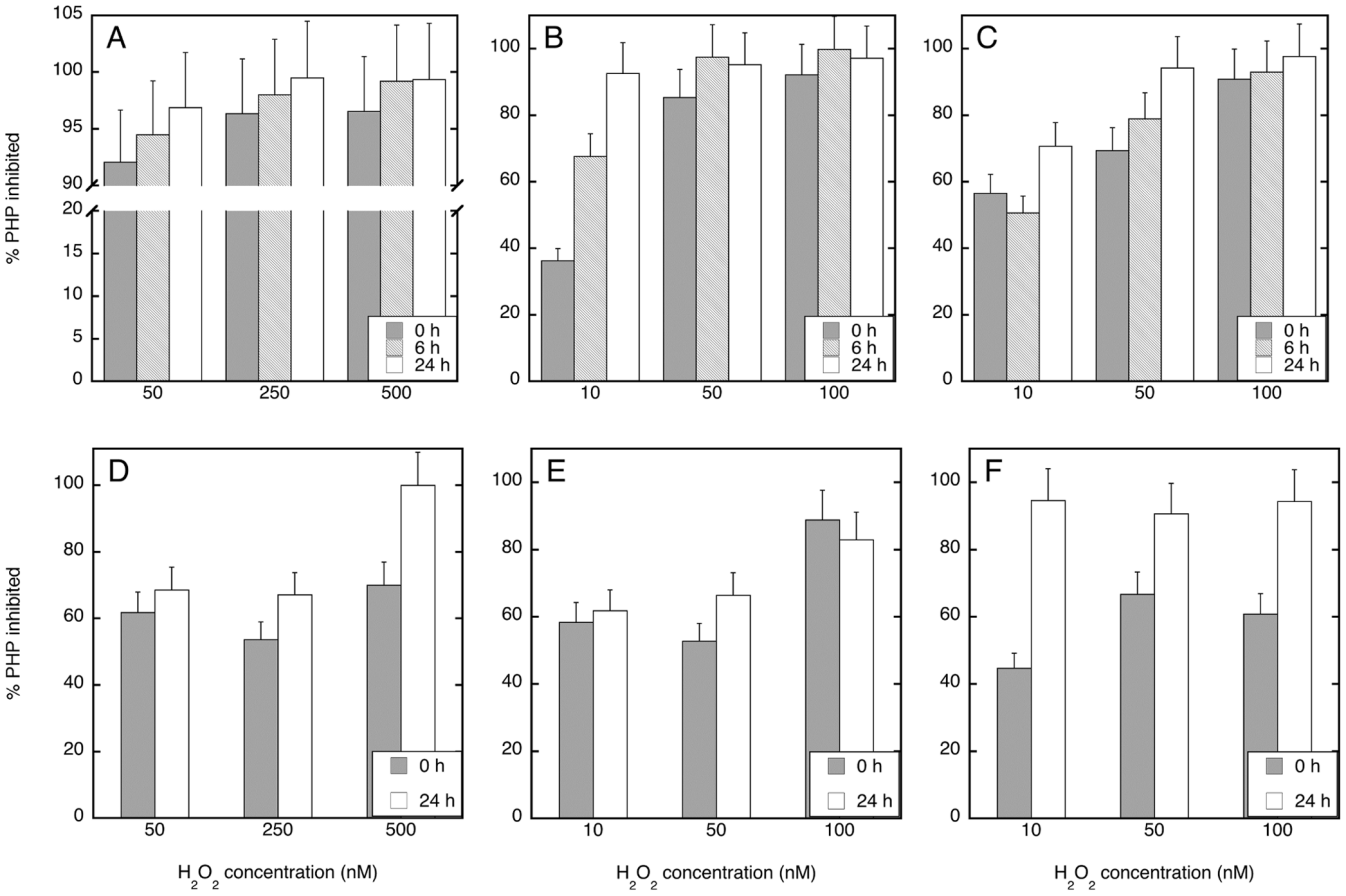
Average (±SD) temporal variation in the percentage of PHP inhibited under different H_2_O_2_ concentrations as compared to the unamended control. PHP was estimated at 100 m (A, B, C) and the oxygen minimum layer (D, E, F) in the three experiments done at Stn. 18 (A, D), 20 (B, E) and 21 (C, F).

**Figure 3:**
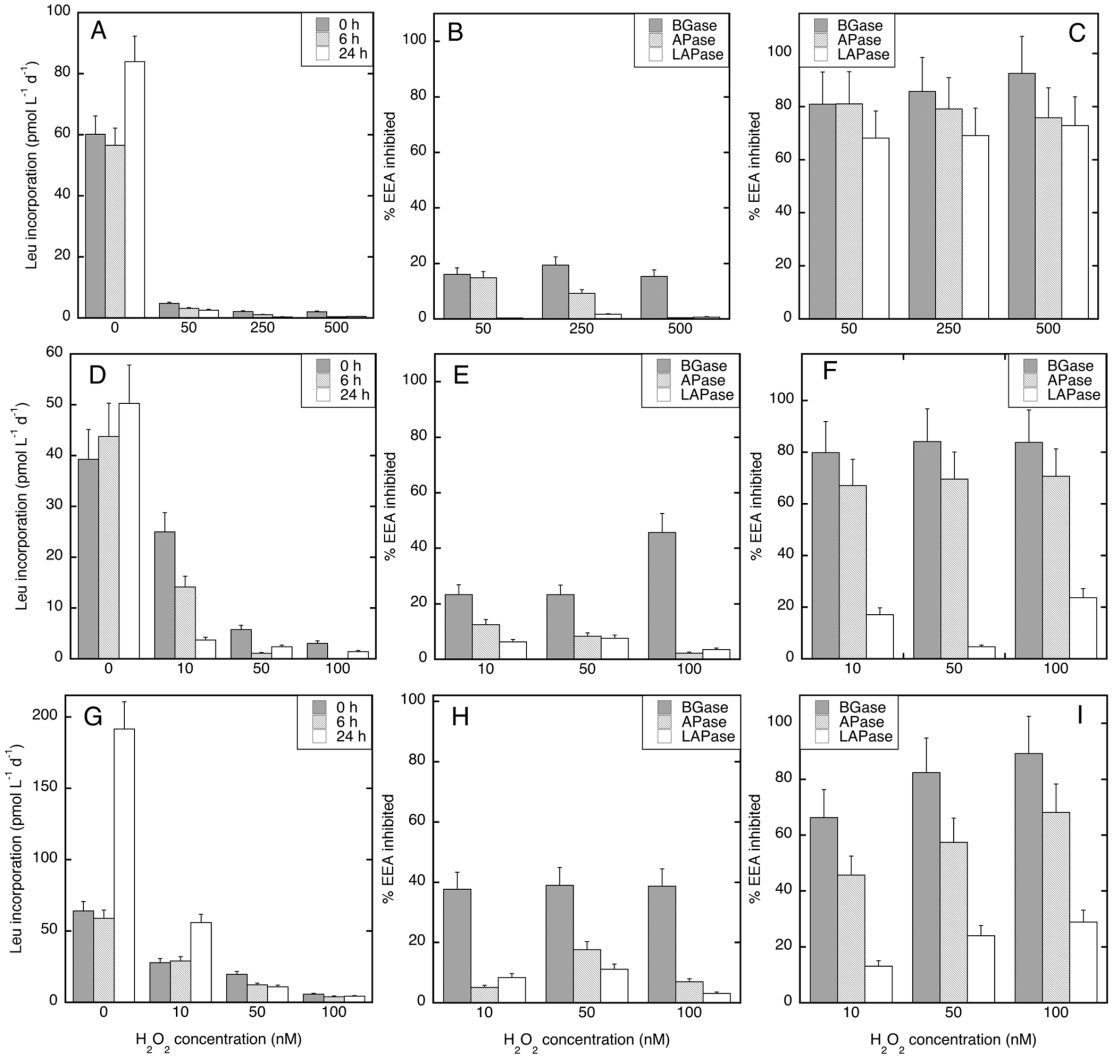
Average (±SD) temporal variation in leucine incorporation rates and extracellular enzymatic activity under different H_2_O_2_ concentrations at 100 m depth. Leucine incorporation rates (A, D, G) were estimated at the start of the experiment, after 6 h and 24 h. The percentages of extracellular enzymatic activities inhibited under different H_2_O_2_ concentrations as compared to the unamended control were estimated at time zero (B, E, H) and after 24 h incubation (C, F, I). Experiments were performed at station 18 (A, B, C), station 20 (D, E, F), and station 21 (G, H, I). BGase: β-glucosidase, APase: alkaline phosphatase, LAPase: leucine aminopeptidase.

**Figure 4:**
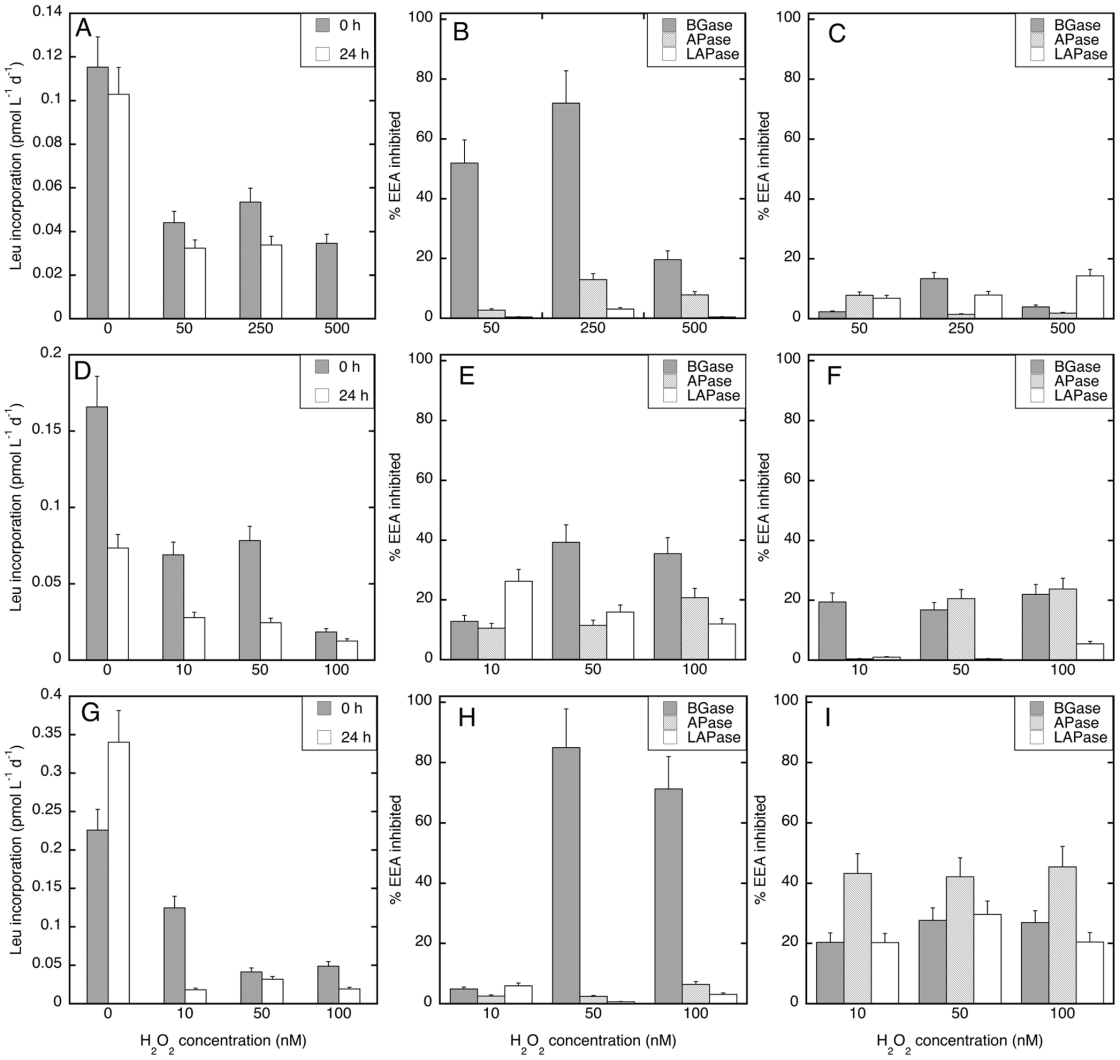
Average (±SD) temporal variation in leucine incorporation rates and extracellular enzymatic activity under different H_2_O_2_ concentrations at the oxygen minimum layer (OML). Leucine incorporation rates (A, D, G) and the percentage of EEA inhibited under different H_2_O_2_ concentrations as compared to the unamended control were estimated at time zero (B, E, H) and after 24 h incubation (C, F, I) in experiment done at St. 18 (A, B, C), St. 20 (D, E, F), and St. 21 (G, H, I). BGase: β-glucosidase, APase: alkaline phosphatase, LAPase: leucine aminopeptidase.

Since short-term impacts of H_2_O_2_ on enzyme activities were detected (Table 1), time course experiments were performed at Stn. 18, 20 and 21 to determine the temporal variations of this inhibitory effect of H_2_O_2_ on both PHP and EEA (Figs. 2, 3, 4). Since already the lowest H_2_O_2_ concentration used in the first set of experiments substantially inhibited the microbial metabolic rates, we applied lower concentrations of H_2_O_2_ in these experiments.

PHP was reduced by 36–100% depending on the H_2_O_2_ concentration and incubation time in both the 100 m layer and OML waters (Fig. 2). This inhibitory effect on PHP augmented with increasing H_2_O_2_ concentrations and incubation time with the strongest impact on PHP at the highest H_2_O_2_ concentrations (i.e., 100–500 nM) after an incubation time of 24 h and the lowest at time zero with 10 nM H_2_O_2_. Significantly stronger (Wilcoxon rank sum test, p<0.005) inhibition of PHP rates towards higher H_2_O_2_ concentrations was found in the second (0 and 6 h incubation) and third (0, 6 and 24 h incubation) experiments in the 100 m layer, and in the first (24 h incubation), second (0 and 24 h incubation) and third (0 h incubation) experiments in the OML waters (Fig. 2). The highest H_2_O_2_ concentration of this set of experiments (i.e., 500 nM) initially reduced PHP by 70% and 97% in the OML and 100 m waters, respectively, and almost completely inhibited PHP at both depths after 24 h (Fig. 2A, D). Interestingly, PHP increased after 24 h only in the unamended treatment (i.e., where H_2_O_2_ was not added) in the 100 m waters experiments (see Fig. 3A, D, G).

In these experiments, the EEA inhibition pattern at time zero was similar to that found in the first set of experiments performed with waters collected at Stn. 6–16 (compare Fig. 3 and 4 to Table 1). Also in the second set of experiments, BGase was generally most strongly inhibited and LAPase was the least inhibited EEA. As for PHP, the inhibition of EEAs due to H_2_O_2_ was stronger after 24 h than at the initial time in all the 100 m experiments (Wilcoxon rank sum test, p<0.001) (Fig. 3). This increased H_2_O_2_-induced inhibition after 24 h was not only found for BGase but also for APase and LAPase, the latter EEAs being reduced by 20–80% (Fig. 3C, F, I). In contrast, less inhibition was found in the OML experiments after 24 h than in the 100 m layer (Wilcoxon rank sum test, p<0.005). In fact, only in one out of the three OML experiments (St. 21; Fig. 4I), the inhibition of APase and LAPase (but not BGase) was stronger after 24 h (Wilcoxon rank sum test, p<0.005) (Fig. 4).

## Discussion

In this study, a major inhibiting effect of H_2_O_2_ on prokaryotic metabolism as deduced from measurements of EEA and PHP was found throughout the Northeast Atlantic water column. Few other studies have investigated the effect of H_2_O_2_ on prokaryotes in aquatic environments, and to the best of our knowledge none have been done in open ocean waters. In our study region, H_2_O_2_ concentrations range between 75–220 nM in surface waters but decrease with depth to 5–10 nM below 100 m depth [6], suggesting that the relative contribution of in situ H_2_O_2_ should be very different between samples collected in the upper mixed layer and the ones from the deep ocean. Previous findings suggest that oxidative stress caused by H_2_O_2_ may become important above the 100 nM level in surface seawater [29], [30]. Our results suggest that H_2_O_2_ concentrations lower than 100 nM still significantly retard prokaryotic metabolism in the subtropical North Atlantic water column reducing EEA and PHP by about 1–85% and 38–96%, respectively (Figs. 2, 3, 4).

We also found that the detrimental impact of H_2_O_2_ on prokaryotic metabolism varies with time depending on the parameter investigated. For instance, although APase and LAPase were not strongly reduced by H_2_O_2_ initially, they were greatly inhibited after 24 h of exposure to H_2_O_2_ (Fig. 3). Recently, it was found that even short-term (∼4 h) exposure to another reactive oxygen species (singlet oxygen) has profound effects on the prokaryotic community composition in a German lake [42]. This implies that reactive oxygen species can have a dual effect on the prokaryotic community: i) directly reducing the metabolic activity of the in situ community, and ii) altering the community structure and thus, indirectly affecting metabolic rates. We cannot confirm that a shift in community composition occurred in our experiments since we did not measure it.

It is also noteworthy that H_2_O_2_ did not reduce all EEAs equally but preferentially inhibited BGase, thereby shaping the spectrum of EEAs. Although there are no previous studies investigating the effect of H_2_O_2_ on EEA rates, the inhibition observed in the EEAs reflects the potential importance of reactive oxygen species such as H_2_O_2_ in affecting the initial step in heterotrophic processing of polymeric organic matter in the ocean. This is critical since the activity of extracellular enzymes largely determines the supply of low molecular weight substrates for direct bacterial uptake [43]. The preferential inhibition of BGase over LAPase (Fig. 3) would result in an increase in the LAPase:BGase ratio. This can be interpreted as an enhanced degradation of protein over polysaccharides [44] which, in turn, ultimately affects the quality and quantity of the organic matter pool available to microbial communities.

Studies investigating the role of H_2_O_2_ in lakes report contrasting results. Studying the effect of photodegradation of humic substances on prokaryotic metabolism in a Swedish lake, Anesio et al. [31] found that H_2_O_2_ concentrations of about 2000–3000 nM were inhibitory for PHP. This range was similar to that formed during UV exposure of aquatic macrophyte leachates (4000–8000 nM H_2_O_2_), suggested to inhibit growth of prokaryotes [45]. However, those H_2_O_2_ levels are around one order of magnitude higher than reported by Xenopoulos and Bird [22] for a Canadian humic lake. These authors found that small amounts of added H_2_O_2_ (<50 nM) inhibited PHP in that lake, and that 100 nM H_2_O_2_ inhibited the PHP by as much as 40%. Although the photochemical production of low-molecular-weight substances from recalcitrant dissolved organic matter can stimulate prokaryotic activity and growth [46], [47], photochemical reactions are known to inhibit prokaryotic activities [48], [49], [50] by the formation of inhibitory substances such as H_2_O_2_ [51], [52], [53], but also by polymerization and condensation of labile compounds [54], [55] and direct mineralization of dissolved organic matter [56], [57]. Thus, these complementary effects of the photolysis of dissolved organic matter on prokaryotic activity might mask the direct effect of H_2_O_2_ on prokaryotic metabolic rates in surface waters. This could potentially explain why in those photochemical studies where H_2_O_2_ concentrations were raised by exposing samples to UV radiation, the inhibitory H_2_O_2_ concentrations were higher compared to experiments where H_2_O_2_ was directly added without exposure to UV radiation.

H_2_O_2_ concentrations have been shown to follow a diel periodicity in oceanic surface waters in our study region, ranging between ∼50–220 nM with increasing concentrations until the mid afternoon [6]. According to our data and assuming that surface water prokaryotes would behave similarly to those at 100 m depth, the range in H_2_O_2_ concentrations in surface waters could provoke a considerable PHP inhibition. Although speculative, our results could then suggest the possibility that the periodicity of PHP observed in the surface waters of the ocean (e.g. [58]), commonly interpreted as UV-induced [59], could be due, at least partly, to the accumulation of H_2_O_2_ in surface waters during daytime.

Taken together, our results suggest a potentially relevant role of oxidative stress affecting bacterioplankton metabolism, reducing prokaryotic growth and the hydrolysis of specific components of the organic matter pool. Reactive oxygen species such as H_2_O_2_ can significantly reduce the amount of organic carbon being channeled through prokaryotes, as well as alter the bioavailability of organic carbon and nutrients via the reduction of PHP and EEA rates and shifts in the EEA spectrum. This influence of oxidative stress could be potentially important throughout the entire oceanic water column where the dark biological production of H_2_O_2_ has been recently shown to be significant [14]. These effects would be even more crucial in specific (micro)environments where elevated concentrations of biologically-produced reactive oxygen species can be expected, like in particle-attached communities. The recent finding of considerable concentrations of hydroperoxides in sinking particles collected in the Arctic Ocean [15] supports this idea. Due to the assumed preferential particle-associated way of life of dark ocean prokaryotes [16], [17], [18], [19], the impact of oxidative stress on this community should be particularly significant. Taking into account the paramount role of open-ocean prokaryotes in the marine biogeochemical cycles, reactive oxygen species such as H_2_O_2_ can have relevant repercussions in marine carbon fluxes.

## Supporting Information

Table S1 Physicochemical characteristics of the stations where hydrogen peroxide enrichment experiments were carried out during the MEDEA cruise in October–November 2011.
(DOC)

Table S2 Response in the microbial extracellular enzymatic activities to all the different H_2_O_2_ concentrations (0, 100, 500, 1000 nM) used in the experiments done with epi- meso- and bathypelagic waters at Stn. 6–16. LAPase: leucine aminopeptidase, APase: alkaline phosphatase, BGase: β-glucosidase.
(DOC)
