# Supplementary material for: Major Effect of Hydrogen Peroxide on Bacterioplankton Metabolism in the Northeast Atlantic
Source: PLoS One. 2013 Apr 12;8(4):e61051. doi: 10.1371/journal.pone.0061051 (PMC3625187; doi:10.1371/journal.pone.0061051)
Supplement: Table S1 — Physicochemical characteristics of the stations where hydrogen peroxide enrichment experiments were carried out during the MEDEA cruise in October–November 2011. (DOC) [file pone.0061051.s001.doc]

Table S1. Physicochemical characteristics of the stations where hydrogen peroxide enrichment experiments were carried out during the MEDEA cruise in October-November 2011.

| Station | Depth (m) | Temp (ºC) | Salinity (psu) | Oxygen (µmol kg^-1^) | PO_4_ (µmol kg^-1^) | Si (µmol kg^-1^) | NO_3_ (µmol kg^-1^) | NO_2_ (µmol kg^-1^) |
| --- | --- | --- | --- | --- | --- | --- | --- | --- |
| 6 | 2790 | 3.2 | 34.93 | 275.7 | 1.18 | 16.64 | 17.69 | 0.02 |
| 6 | 992 | 9.6 | 35.50 | 189.9 | 1.13 | 9.41 | 18.17 | 0.03 |
| 6 | 100 | 13.0 | 35.76 | 240.7 | 0.50 | 2.85 | 8.29 | 0.04 |
| 7 | 2787 | 3.0 | 34.94 | 268.2 | 1.23 | 22.10 | 18.67 | 0.02 |
| 7 | 909 | 10.5 | 35.82 | 185.3 | 1.03 | 8.96 | 17.05 | 0.02 |
| 7 | 100 | 14.0 | 35.90 | 236.1 | 0.37 | 1.83 | 6.16 | 0.06 |
| 8 | 2790 | 3.0 | 34.95 | 261.0 | 1.29 | 25.92 | 19.37 | 0.02 |
| 8 | 903 | 10.6 | 35.83 | 181.1 | 1.04 | 9.08 | 17.22 | 0.02 |
| 8 | 100 | 15.7 | 36.15 | 226.6 | 0.18 | 1.14 | 2.85 | 0.12 |
| 9 | 2789 | 3.1 | 34.95 | 262.3 | 1.26 | 23.45 | 19.18 | 0.02 |
| 9 | 619 | 11.5 | 35.59 | 204.4 | 0.86 | 5.59 | 14.40 | 0.02 |
| 9 | 100 | 16.6 | 36.27 | 225.6 | 0.11 | 1.07 | 2.04 | 0.16 |
| 10 | 2787 | 2.8 | 34.94 | 256.4 | 1.34 | 30.38 | 20.27 | 0.03 |
| 10 | 709 | 10.4 | 35.67 | 183.3 | 1.07 | 8.87 | 17.59 | 0.03 |
| 10 | 100 | 15.5 | 36.12 | 225.1 | 0.25 | 1.46 | 3.98 | 0.06 |
| 12 | 2785 | 2.9 | 34.95 | 258.2 | 1.33 | 28.38 | 20.10 | 0.02 |
| 12 | 710 | 10.6 | 35.49 | 178.1 | 1.12 | 8.63 | 18.57 | 0.03 |
| 12 | 100 | 18.4 | 36.62 | 218.0 | 0.04 | 0.82 | 1.09 | 0.08 |
| 13 | 2784 | 3.0 | 34.96 | 256.9 | 1.33 | 28.40 | 20.06 | 0.02 |
| 13 | 806 | 10.1 | 35.49 | 177.0 | 1.17 | 9.83 | 19.21 | 0.02 |
| 13 | 100 | 19.2 | 36.67 | 232.9 | 0.01 | 0.73 | 0.01 | 0.01 |
| 14 | 2783 | 2.9 | 34.96 | 253.6 | 1.38 | 30.93 | 20.82 | 0.02 |
| 14 | 849 | 9.3 | 35.36 | 168.1 | 1.34 | 11.92 | 21.62 | 0.03 |
| 14 | 100 | 20.3 | 36.84 | 232.7 | 0.01 | 0.56 | 0.00 | 0.02 |
| 16 | 2786 | 2.9 | 34.96 | 248.9 | 1.45 | 32.68 | 21.50 | 0.02 |
| 16 | 779 | 9.2 | 35.33 | 143.4 | 1.56 | 13.50 | 24.74 | 0.02 |
| 16 | 100 | 22.0 | 37.26 | 227.6 | 0.02 | 0.50 | 0.03 | 0.01 |
| 18 | 2782 | 2.9 | 34.96 | 253.2 | 1.40 | 32.44 | 21.11 | 0.02 |
| 18 | 900 | 8.3 | 35.32 | 160.7 | 1.52 | 14.72 | 24.01 | 0.02 |
| 18 | 100 | 21.4 | 37.20 | 227.8 | 0.01 | 0.54 | 0.03 | 0.01 |
| 20 | 2787 | 3.0 | 34.97 | 253.8 | 1.37 | 29.90 | 20.60 | 0.02 |
| 20 | 747 | 10.5 | 35.50 | 218.9 | 1.29 | 14.56 | 20.10 | 0.01 |
| 20 | 100 | 19.8 | 36.88 | 231.6 | 0.01 | 0.57 | 0.01 | 0.02 |
| 21 | 2783 | 3.0 | 34.97 | 251.6 | 1.44 | 36.82 | 21.69 | 0.02 |
| 21 | 831 | 10.1 | 35.52 | 173.5 | 1.20 | 9.79 | 19.61 | 0.01 |
| 21 | 100 | 18.7 | 36.55 | 244.2 | 0.01 | 0.57 | 0.05 | 0.01 |
