# Supplementary material for: Major Effect of Hydrogen Peroxide on Bacterioplankton Metabolism in the Northeast Atlantic
Source: PLoS One. 2013 Apr 12;8(4):e61051. doi: 10.1371/journal.pone.0061051 (PMC3625187; doi:10.1371/journal.pone.0061051)
Supplement: Table S2 — Response in the microbial extracellular enzymatic activities to all the different H2O2 concentrations (0, 100, 500, 1000 nM) used in the experiments done with epi- meso- and bathypelagic waters at Stn. 6–16. LAPase: leucine aminopeptidase, APase: alkaline phosphatase, BGase: β-glucosidase. (DOC) [file pone.0061051.s002.doc]

Table S2. Response in the microbial extracellular enzymatic activities to all the different H_2_O_2_ concentrations (0, 100, 500, 1000 nM) used in the experiments done with epi- meso- and bathypelagic waters at Stn. 6-16. LAPase: leucine aminopeptidase, APase: alkaline phosphatase, BGase: β-glucosidase.

| Station | Depth (m) | H_2_O_2_ (nM) | LAPase (nM h^-1^) | APase (nM h^-1^) | BGase (nM h^-1^) |
| --- | --- | --- | --- | --- | --- |
| 6 | 100 | 0 | 6.0 | 0.61 | 0.122 |
| 6 | 100 | 100 | 6.0 | 0.61 | 0.089 |
| 6 | 100 | 500 | 6.1 | 0.62 | 0.003 |
| 6 | 100 | 1000 | 5.7 | 0.61 | 0.004 |
| 6 | 990 | 0 | 4.2 | 0.43 | 0.063 |
| 6 | 990 | 100 | 4.3 | 0.44 | 0.060 |
| 6 | 990 | 500 | 4.0 | 0.43 | 0.013 |
| 6 | 990 | 1000 | 3.3 | 0.37 | 0.041 |
| 6 | 2790 | 0 | 4.4 | 0.64 | 0.252 |
| 6 | 2790 | 100 | 4.1 | 0.61 | 0.172 |
| 6 | 2790 | 500 | 3.8 | 0.54 | 0.046 |
| 6 | 2790 | 1000 | 4.0 | 0.50 | 0.089 |
| 7 | 100 | 0 | 4.4 | 1.36 | 0.037 |
| 7 | 100 | 100 | 4.2 | 1.10 | 0.016 |
| 7 | 100 | 500 | 3.8 | 0.63 | 0.006 |
| 7 | 100 | 1000 | 4.2 | 0.61 | 0.019 |
| 7 | 909 | 0 | 2.6 | 0.59 | 0.095 |
| 7 | 909 | 100 | 2.5 | 0.57 | 0.075 |
| 7 | 909 | 500 | 2.4 | 0.45 | 0.056 |
| 7 | 909 | 1000 | 2.2 | 0.51 | 0.068 |
| 7 | 2785 | 0 | 0.6 | 0.47 | 0.090 |
| 7 | 2785 | 100 | 0.4 | 0.46 | 0.065 |
| 7 | 2785 | 500 | 0.3 | 0.43 | 0.070 |
| 7 | 2785 | 1000 | 0.4 | 0.48 | 0.081 |
| 8 | 100 | 0 | 5.3 | 0.35 | 0.084 |
| 8 | 100 | 100 | 4.9 | 0.33 | 0.006 |
| 8 | 100 | 500 | 5.1 | 0.34 | 0 |
| 8 | 100 | 1000 | 4.6 | 0.36 | 0 |
| 8 | 903 | 0 | 3.0 | 0.47 | 0.054 |
| 8 | 903 | 100 | 2.8 | 0.44 | 0.051 |
| 8 | 903 | 500 | 3.0 | 0.52 | 0.032 |
| 8 | 903 | 1000 | 2.9 | 0.57 | 0.020 |
| 8 | 2790 | 0 | 2.2 | 0.49 | 0.111 |
| 8 | 2790 | 100 | 2.2 | 0.37 | 0.095 |
| 8 | 2790 | 500 | 2.1 | 0.41 | 0.094 |
| 8 | 2790 | 1000 | 2.1 | 0.49 | 0.067 |
| 9 | 100 | 0 | 6.6 | 0.47 | 0.128 |
| 9 | 100 | 100 | 6.0 | 0.44 | 0.077 |
| 9 | 100 | 500 | 5.3 | 0.42 | 0.076 |
| 9 | 100 | 1000 | 5.8 | 0.40 | 0.073 |
| 9 | 619 | 0 | 3.0 | 0.41 | 0.033 |
| 9 | 619 | 100 | 2.9 | 0.41 | 0.027 |
| 9 | 619 | 500 | 2.9 | 0.41 | 0.029 |
| 9 | 619 | 1000 | 2.9 | 0.40 | 0.033 |
| 9 | 2789 | 0 | 1.7 | 0.46 | 0.089 |
| 9 | 2789 | 100 | 1.6 | 0.33 | 0 |
| 9 | 2789 | 500 | 1.5 | 0.37 | 0 |
| 9 | 2789 | 1000 | 1.5 | 0.37 | 0.068 |
| 10 | 100 | 0 | 3.7 | 0.53 | 0.241 |
| 10 | 100 | 100 | 3.6 | 0.57 | 0.098 |
| 10 | 100 | 500 | 3.8 | 0.54 | 0.166 |
| 10 | 100 | 1000 | 3.8 | 0.46 | 0.173 |
| 10 | 709 | 0 | 0.5 | 0.49 | 0.134 |
| 10 | 709 | 100 | 0.4 | 0.48 | 0.135 |
| 10 | 709 | 500 | 0.4 | 0.50 | 0.086 |
| 10 | 709 | 1000 | 0.4 | 0.42 | 0.111 |
| 10 | 2787 | 0 | 0.3 | 0.31 | 0.051 |
| 10 | 2787 | 100 | 0.2 | 0.23 | 0.025 |
| 10 | 2787 | 500 | 0.3 | 0.26 | 0.029 |
| 10 | 2787 | 1000 | 0.2 | 0.23 | 0.034 |
| 12 | 100 | 0 | 7.2 | 0.56 | 0.067 |
| 12 | 100 | 100 | 6.4 | 0.55 | 0.036 |
| 12 | 100 | 500 | 6.5 | 0.49 | 0.013 |
| 12 | 100 | 1000 | 6.9 | 0.46 | 0.051 |
| 12 | 710 | 0 | 2.7 | 0.55 | 0.124 |
| 12 | 710 | 100 | 2.6 | 0.52 | 0.084 |
| 12 | 710 | 500 | 2.4 | 0.52 | 0.053 |
| 12 | 710 | 1000 | 2.6 | 0.56 | 0.050 |
| 12 | 2786 | 0 | 1.6 | 0.54 | 0.099 |
| 12 | 2786 | 100 | 1.5 | 0.53 | 0.080 |
| 12 | 2786 | 500 | 1.5 | 0.50 | 0.075 |
| 12 | 2786 | 1000 | 1.6 | 0.52 | 0.080 |
| 13 | 100 | 0 | 7.1 | 0.90 | 0.104 |
| 13 | 100 | 100 | 6.8 | 0.99 | 0.037 |
| 13 | 100 | 500 | 6.8 | 0.88 | 0.007 |
| 13 | 100 | 1000 | 6.2 | 0.88 | 0.054 |
| 13 | 806 | 0 | 1.0 | 0.69 | 0.106 |
| 13 | 806 | 100 | 0.9 | 0.63 | 0.077 |
| 13 | 806 | 500 | 0.9 | 0.63 | 0.086 |
| 13 | 806 | 1000 | 0.9 | 0.65 | 0.088 |
| 13 | 2784 | 0 | 1.2 | 0.43 | 0.101 |
| 13 | 2784 | 100 | 1.1 | 0.38 | 0.060 |
| 13 | 2784 | 500 | 1.0 | 0.38 | 0.046 |
| 13 | 2784 | 1000 | 1.0 | 0.39 | 0.059 |
| 14 | 100 | 0 | 11.6 | 0.81 | 0.056 |
| 14 | 100 | 100 | 11.1 | 0.75 | 0.039 |
| 14 | 100 | 500 | 11.0 | 0.67 | 0.025 |
| 14 | 100 | 1000 | 10.9 | 0.66 | 0.033 |
| 14 | 849 | 0 | 3.1 | 0.68 | 0.153 |
| 14 | 849 | 100 | 2.6 | 0.57 | 0.110 |
| 14 | 849 | 500 | 2.4 | 0.62 | 0.102 |
| 14 | 849 | 1000 | 2.9 | 0.56 | 0.141 |
| 14 | 2783 | 0 | 2.2 | 0.24 | 0.083 |
| 14 | 2783 | 100 | 2.1 | 0.18 | 0.033 |
| 14 | 2783 | 500 | 2.0 | 0.23 | 0.035 |
| 14 | 2783 | 1000 | 2.0 | 0.22 | 0.017 |
| 16 | 100 | 0 | 11.3 | 1.28 | 0.082 |
| 16 | 100 | 100 | 10.7 | 1.25 | 0.059 |
| 16 | 100 | 500 | 10.0 | 0.93 | 0.054 |
| 16 | 100 | 1000 | 9.6 | 0.86 | 0.048 |
| 16 | 779 | 0 | 3.5 | 0.48 | 0.093 |
| 16 | 779 | 100 | 3.4 | 0.45 | 0.060 |
| 16 | 779 | 500 | 3.2 | 0.44 | 0.045 |
| 16 | 779 | 1000 | 3.4 | 0.46 | 0.057 |
| 16 | 2786 | 0 | 0.9 | 0.30 | 0.033 |
| 16 | 2786 | 100 | 1.0 | 0.30 | 0.012 |
| 16 | 2786 | 500 | 0.9 | 0.31 | 0.023 |
| 16 | 2786 | 1000 | 1.0 | 0.31 | 0.043 |
